# *Polymorphospora lycopeni* A560, a new strain capable of producing lycopene, and its optimal fermentation and extraction conditions

**DOI:** 10.3389/fmicb.2025.1723758

**Published:** 2026-01-22

**Authors:** Xiaomin Duan, Shilong Li, Yan Li, Zihao Yang, Xiumin Zhang

**Affiliations:** 1 College of Life Sciences, Hebei University, Baoding, Hebei, China; 2 Key Laboratory of Microbial Diversity Research and Application of Hebei Province, Baoding, Hebei, China; 3 Engineering Research Center of Microbial Breeding and Conservation, Baoding, Hebei, China

**Keywords:** extracting conditions, lycopene, optimization of culture medium, *Polymorphospora lycopeni* A560, *Va/Vm* ratios

## Abstract

**Background:**

Lycopene, a vital antioxidant, can reduce oxidative damage to cells caused by reactive oxygen, prevent cancer, lower blood cholesterol levels, and mitigate cardiovascular diseases. We screened a lycopene-producing strain of the genus *Polymorphospora*, designated A560, and discovered that its metabolic pathway lacks the lycopene *β*-cyclase gene *crtY*, which is responsible for converting lycopene into *β*-carotene. The absence of *crtY* alleviates the challenges associated with the addition of a cyclooxygenase inhibitor, which previously hindered the large-scale accumulation of lycopene in *Blakeslea trispora*.

**Method:**

This study was conducted to enhance lycopene production in strain A560 through a multi-stage optimization strategy, focusing on improving extraction efficiency and optimizing culture medium components. The extraction conditions were optimized by testing different solvent systems (acetone/n-hexane/ethanol), extraction temperatures, and durations. Subsequently, a uniform design (UD) was applied to systematically optimize the composition of the culture medium to maximize lycopene yield.

**Results:**

The extraction conditions were systematically optimized. A crushing duration of 4 min was identified as optimal for complete cell disruption. The most efficient solvent system was determined to be n-hexane/ethanol (2:1, *v/v*) at 60 °C, which yielded 62.05 ± 4.68 mg/L of lycopene—a 37.13% increase over extraction with acetone at room temperature (45.25 ± 0.98 mg/L). Subsequent medium optimization through a uniform design revealed that soluble starch and glycerol were critical components. Cultivation in the theoretically optimized medium predicted by the model resulted in a lycopene yield of 142.54 ± 8.58 mg/L. Finally, regulating the oxygen supply by adjusting the flask’s volume of air to a medium volume (*Va/Vm*) ratio proved crucial. The highest lycopene production of 201.44 ± 6.23 mg/L was achieved at a *Va/Vm* ratio of 1.5, which marks a 224.64% improvement over the yield obtained after the initial extraction optimization (62.05 ± 4.68 mg/L).

**Conclusion:**

The extraction and fermentation processes for lycopene production in *Polymorphospora* sp. A560 were successfully optimized, resulting in significantly enhanced yields. These results demonstrate that strain A560 is a promising microbial resource for the fermentative production of lycopene.

## 1 Introduction

Lycopene is a fat-soluble natural pigment and is an important member of the carotenoid family (Stahl and Sies, 1996). It was first isolated from processed tomatoes, often referred to as the “gold” of tomatoes. As a powerful antioxidant, lycopene can mitigate oxidative damage caused by reactive oxygen species in cells, reduce radiation damage, and help to prevent cancer. Additionally, it contributes to the regulation of blood lipids, lowering cholesterol levels, and preventing cardiovascular and cerebrovascular diseases. Lycopene has also been shown to improve male fertility and decrease the incidence of prostate-related conditions (Stahl and Sies, 1996; Cheng et al., 2020; Ilic and Misso, 2012; Kulawik et al., 2023; Li et al., 2021; Müller et al., 2016; Mordente et al., 2011; Stinco et al., 2013; Sharoni et al., 2012; Zhao et al., 2018). Recent studies suggest that lycopene may enhance the body’s physiological defense against the COVID-19 infection (Barciszewska, 2021; Khongthaw et al., 2022). Consequently, lycopene is increasingly being incorporated into functional foods, pharmaceuticals, and cosmetics, demonstrating promising commercial potential (Wei et al., 2023).

Lycopene is highly lipophilic and virtually insoluble in ethanol, methanol, and water (Saini et al., 2015). Unlike the majority of other carotenoids, lycopene has a linear structure with a molecular formula of C_40_H_56_ and a molecular weight of 536.8557 Da (Li et al., 2020). Microbial fermentation has become a pivotal platform for sustainable lycopene production. Compared with plant extraction (Li et al., 2020), it enables the “de-agriculturalization” of manufacturing by transferring output from land-intensive cultivation to efficient bioreactors. Current *Saccharomyces cerevisiae* strains reach titers of 8.15 g/L (Zhou et al., 2023), allowing a single 120-m^3^ fermenter to yield nearly one ton of high-purity lycopene per batch—an output that would otherwise require thousands of hectares of farmland and substantial water inputs. This approach eliminates dependence on climatic and agronomic uncertainties, thereby ensuring a stable supply chain. In contrast to chemical synthesis (Ernst, 2002; Reynaud et al., 2011), microbial pathways offer “nature-identical” products, as strains such as *Blakeslea trispora* inherently favor the formation of all-trans lycopene (Schieber and Carle, 2005), avoiding stereochemical complexity and synthetic by-products. This results in superior safety and bioactivity, making microbially derived lycopene particularly suitable for pharmaceutical and premium nutraceutical applications. Therefore, microbial fermentation is increasingly recognized as a green, sustainable, and high-quality route for lycopene production (Ward et al., 2018), aligning closely with modern demands for environmentally responsible and clean-label manufacturing.

To date, many microorganisms have been reported to produce lycopene, including bacteria, fungi, and algae, such as *Dietzia natronolimnae* (Nasri Nasrabadi and Razavi, 2010), *Blakeslea trispora* (Nasri Nasrabadi and Razavi, 2010; Choudhari et al., 2008; Liu and Shen, 2004; Liu et al., 2012; Mehta et al., 2003; Qiang et al., 2014), *Rhodotorula glutinis* (Squina and Mercadante, 2005), *R. rubra* (Squina and Mercadante, 2005), *Dunaliella salina* (Fazeli et al., 2009), and *Haematococcus pluvialis* (Vidhyavathi et al., 2009). Among these lycopene-producing strains, *Blakeslea trispora* is the only one that has achieved industrial production. However, it has the disadvantage of a complex fermentation process and requires the inhibition of cyclase activity in the lycopene synthesis pathway to produce high-purity lycopene (Wang et al., 2023). The high cost of cyclase inhibitors limits their application in lycopene production, and there are food safety risks associated with using cyclase inhibitors as additives. Consequently, research on microbial fermentation for lycopene production focuses on searching for safe, reliable, and cost-effective lycopene-producing strains that can be further optimized to enhance lycopene yield through optimizing culture conditions and extraction methods.

We screened a lycopene-producing strain, *Polymorphospora lycopeni* A560 (Liu et al., 2024), which was the first identified lycopene-producing member from the genus *Polymorphospora*. The strain A560 does not need the addition of expensive and polluting cyclase inhibitors during fermentation, and it is easy to accumulate and purify lycopene. Additionally, this strain offers the advantages of a simple fermentation medium and a short fermentation period. By optimizing the carbon source and dissolved oxygen levels, we identified the optimal medium and culture conditions for lycopene production by the strain A560. Furthermore, we established the ideal conditions for extracting lycopene from the fermentation products of the strain A560 and ultimately enhanced the lycopene yield. This research lays the groundwork for the microbial industrial production of lycopene.

## 2 Materials and methods

### 2.1 Strain and initial fermentation culture

The strain *Polymorphospora lycopeni* A560 utilized in this study was isolated previously by members of our laboratory (Liu et al., 2024).

The strain A560 was inoculated into 100 mL of seed medium (soluble starch: 10 g/L, glucose: 10 g/L, peptone: 8 g/L, NaCl: 3 g/L, K_2_HPO_4:_ 1 g/L, MgSO_4_: 1 g/L, pH 7–7.4), which was incubated at 28 °C for 7 days with shaking at 180 rpm to obtain seed cultures. Then, the seed culture was transferred to a 250-mL conical flask containing 100 mL of ISP4 medium (soluble starch: 10 g/L, MgSO_4˙_7H_2_O: 1 g/L, K_2_HPO_4_: 1 g/L, NaCl: 1 g/L, CaCO_3_: 2 g/L, (NH_4_)_2_SO_4_ 2 g/L, trace salt solution: 1 mL/L, pH: 7.0–7.4), with an inoculum concentration of 2%.

Trace salt solution contains FeSO_4˙_7H_2_O (0.1 g/L), MnCl_2˙_4H_2_O (0.1 g/L), and ZnSO_4˙_7H_2_O (0.1 g/L).

### 2.2 Optimization of extraction conditions of lycopene

#### 2.2.1 Optimization of extraction time

The strain A560 was inoculated into ISP4 medium using the previously described method and fermented at 28 °C with shaking at 180 rpm for 7 days (Liu et al., 2024). After that, the mycelia were collected by centrifugation at 4 °C, 6,000 rpm for 10 min and then were lyophilized under vacuum at −50 °C for 48 h. The lyophilized mycelia were weighed to determine the biomass and were stored at −80 °C. In total, 10 mg of lyophilized bacterial cells was precisely weighed into a 1.5-mL grinding tube and mixed with 0.5 mL of analytical-grade acetone along with 0.5 g of glass beads (0.5 mm in diameter) for homogenization. The tube was then placed into a biological sample homogenizer (PRECELLYS-24, Bertin). The disruption procedure was set to 4,500 rpm, with each cycle consisting of 30 s of homogenization followed by a 10-s pause for cooling. Cycling was continued until the total designated extraction time was reached, covering eight time points from 1 to 8 min, with three replicates each. After homogenization, the mixture—comprising cell debris, solvent, and lycopene—was filtered through a 0.22-μm organic solvent-resistant filter membrane, and the filtrate was collected. The residue was repeatedly washed with acetone until the eluate became colorless. All filtrates were combined and adjusted to a final volume of 5 mL with acetone. The resulting solution was stored in amber vials to prevent light-induced degradation.

The lycopene content, which refers to the lycopene titer [mg/L] per unit volume of culture medium in this study, was measured using spectrophotometry at a wavelength of 502 nm. First, a standard curve of lycopene was established to create a linear regression equation that relates absorbance to mass concentration. Next, analytically pure acetone was used as a blank control to measure the absorbance of the extracts. The lycopene yield of strain A560 after fermentation was then calculated using the lycopene standard curve. Additionally, the optimal crushing time of mycelia was determined.

#### 2.2.2 Extraction reagents and temperature

**Figure 1:**
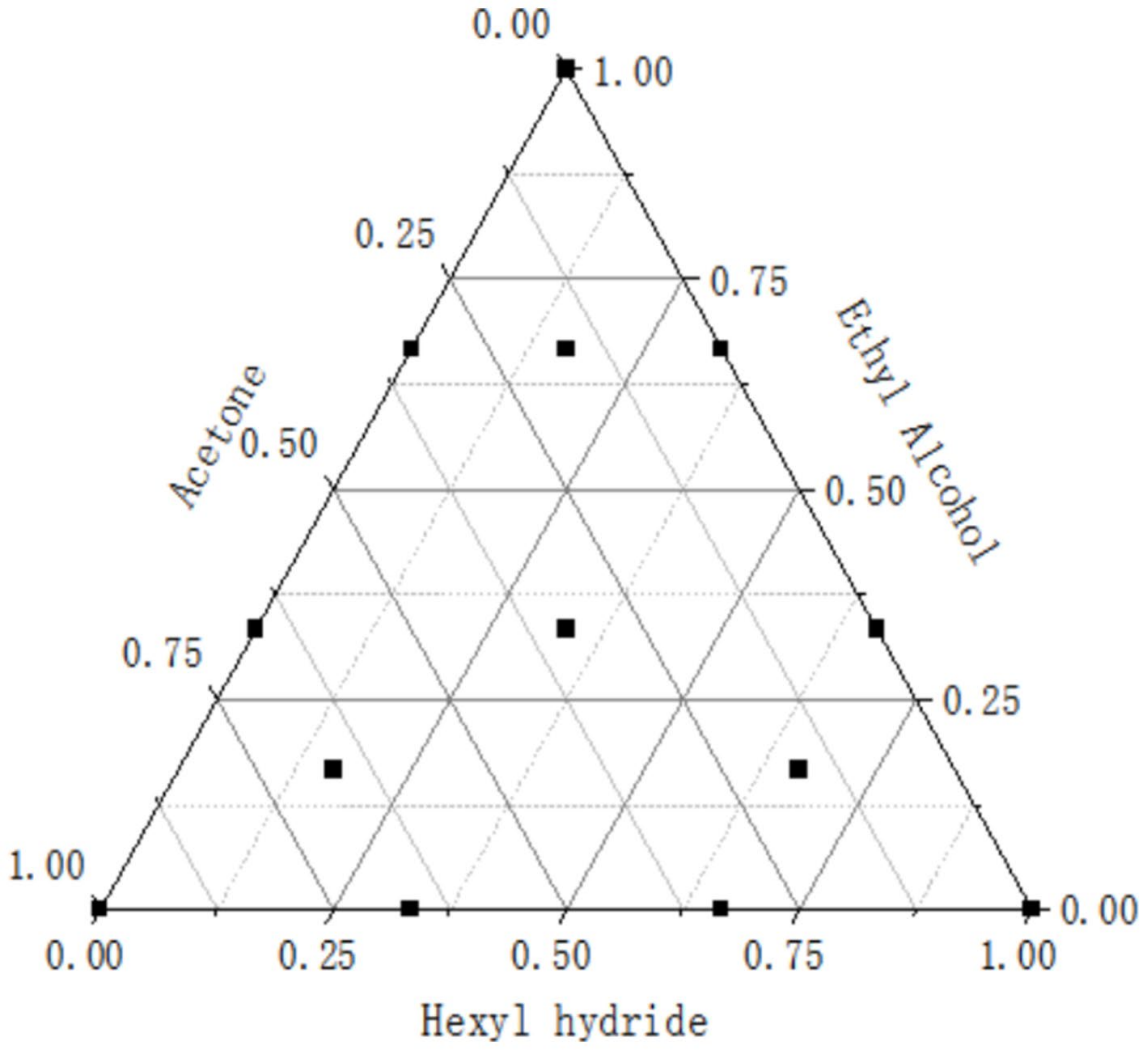
Augmented simplex centroid design for the study of ternary solvent mixtures.

Traditional lycopene extraction typically uses organic solvents (Jiang et al., 2024). Three organic reagents—n-hexane, anhydrous ethanol, and acetone—were selected in this study for the augmented simplex centripetal design (ASCD) to investigate the effects of the composition of solvent mixtures on lycopene recovery (Zuorro, 2020). The ASCD consisted of a {3,3} simplex lattice, with nine equispaced points on the perimeter of the triangle and four points in the internal region (Figure 1). The points indicate 13 different solvent formulations in which the reagents were combined according to different volume ratios. Water baths were maintained at temperatures of 20 °C, 40 °C, and 60 °C for 1 h, respectively, with each condition replicated in triplicate to identify the optimal extraction parameters.

### 2.3 Media optimization

#### 2.3.1 Single-factor experiments

In this study, ISP4 medium served as the base culture medium, and five carbon sources were selected: soluble starch, glucose, maltose, sucrose, and xylan. For each carbon source, five concentrations—5 g/L, 10 g/L, 15 g/L, 20 g/L, and 25 g/L—were used. The corresponding culture media were prepared. They were then inoculated and cultured as previously described. The optimized method was used to extract lycopene and determine the yield. Data are presented as mean ± SD from three independent experiments.

The effects of glycerol on lycopene production were investigated by adding various concentrations (20, 40, 60, 80, and 100 mL/L) of glycerol to the medium prepared with the optimal carbon source.

#### 2.3.2 Optimization of a uniform design

Eight factors, namely soluble starch, MgSO_4_·7H_2_O, K_2_HPO_4_, NaCl, CaCO_3_, (NH_4_)_2_SO_4_, trace salt solution, and glycerol, were set as the independent variables X_1_ to X_8_, respectively, while the lycopene yield was considered the dependent variable Y. A uniform design (UD) was conducted using the software DPS 9.01 to optimize the proportion of each component in the medium for the U9 (9^8^) test, as detailed in Table 1. According to the above methods, the corresponding medium was prepared and inoculated to determine the lycopene yield of the strain A560 after fermentation. The quadratic polynomial stepwise regression was used to analyze the experimental results, the regression equation was verified, and the theoretical optimal medium formula was finally determined. Data are presented as mean ± SD from three independent experiments.

**Table 1:** Optimized UD of fermentation medium for lycopene production by the strain A560.

Groups	Factors							
	X_1_	X_2_	X_3_	X_4_	X_5_	X_6_	X_7_	X_8_
N_1_	4	2	3	2	6	2	8	3
N_2_	2	9	2	4	5	6	1	4
N_3_	5	1	8	8	4	7	3	1
N_4_	8	3	1	5	2	9	6	6
N_5_	9	8	7	6	9	5	7	2
N_6_	7	5	4	7	7	1	2	9
N_7_	1	6	5	9	3	4	9	7
N_8_	6	7	6	1	1	3	4	5
N_9_	3	4	9	3	8	8	5	8

#### 2.3.3 Validation experiments

The strain A560 was cultured with the theoretical optimum medium and the ISP4 medium, and the dry biomass and lycopene yield were measured. The data are presented as mean ± SD from three independent experiments.

### 2.4 Optimization of culture conditions

Referring to the method described by Nanou and Roukas (2011), the strain A560 was inoculated in 500-mL conical flasks containing 50, 100, 150, 200, and 250 mL of the optimal media, respectively, to achieve *Va/Vm* ratios of 9.0, 4.0, 2.3, 1.5, and 1.0, where Va represents the volume of air in the conical flasks and Vm denotes the total volume. According to the above method, the strain A560 was fermented, and lycopene was extracted and analyzed. The data are presented as mean ± SD from three independent experiments.

### 2.5 Statistical analysis

The data were statistically analyzed by the software package IBM SPSS 20 (SPSS). Data are presented as mean ± SD. Means and SDs were determined through descriptive statistics. The data were subjected to an analysis of variance (ANOVA) to determine a significant difference between the overall groups and Duncan’s multiple comparisons to determine if the difference in means between the groups was substantial. A *p-*value of <0.05 was considered statistically significant between the groups.

## 3 Results and discussion

### 3.1 Optimization of extraction conditions

#### 3.1.1 Optimization of extraction time

**Figure 2:**
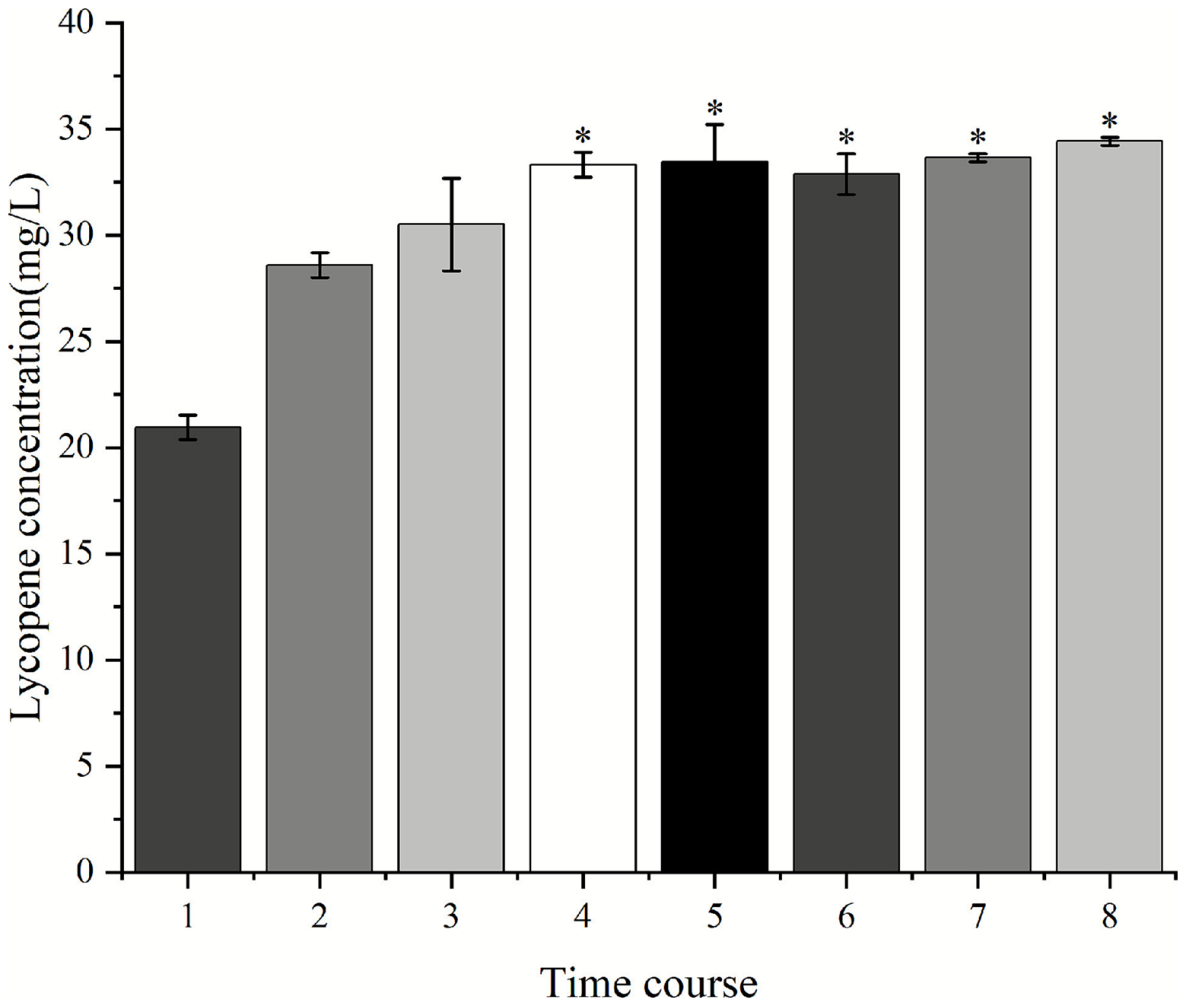
Lycopene yield extracted from strain A560 using acetone as the sole solvent across a time course of 1–8 min. Data are presented as mean ± SD from three independent experiments. ^⁎^*p* < 0.05, significant differences were determined using Duncan’s new multiple range test.

The degree of cell breaking is a crucial factor influencing the extraction efficiency of lycopene (Coelho et al., 2022; Dhakane-Lad and Kar, 2021; Oberoi and Sogi, 2017). This study investigated the impact of crushing duration on the yield of lycopene obtained through extraction. It was shown that crushing time has a significantly positive effect on lycopene yield (Figure 2), with the lycopene yield increasing with crushing time during the first 4 min, reaching a peak of 33.33 mg/L at 4 min. At this point, there was no noticeable orange-red color in the residue after extraction, indicating that the extraction of lycopene was sufficient. When the crushing time was more than 4 min, the lycopene yield was not significantly increased.

#### 3.1.2 Optimization of extraction reagents and temperature

**Figure 3:**
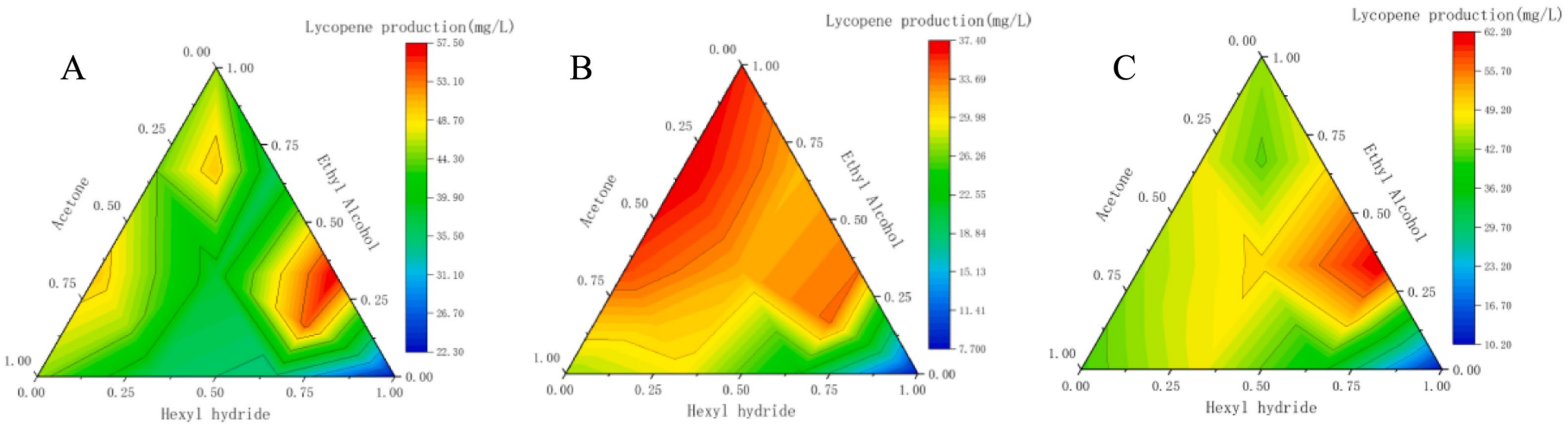
Yield variation of lycopene extracted by different ratios of *n*-hexane–acetone–ethanol at three water bath temperatures: **(A)** 20 °C; **(B)** 40 °C; **(C)** 60 °C.

Due to the heat sensitivity of lycopene, the choice of temperature significantly affects the extraction efficiency of this compound (Dhakane-Lad and Kar, 2021; Oberoi and Sogi, 2017). A contour map was plotted according to the data to illustrate the variation in yield at three different temperatures for various ratios of the three solvents (Figure 3). It was observed that 20–60 °C favored the extraction of lycopene.

High lycopene yield was achieved at the temperatures of 20 °C and 60 °C with a corresponding solvent ratio of n-hexane:ethanol equal to 2:1 (Figures 3A,C). However, the lycopene yield decreased when the temperature was 40 °C (Figure 3B), likely due to the increase of lycopene oleoresin extraction (Zuorro, 2020). Therefore, the optimal solvent formulation was n-hexane:ethanol (2:1), and the optimal temperature was 60 °C, which resulted in the highest lycopene yield of 62.05 ± 4.68 mg/L. N-hexane demonstrates a strong affinity for lycopene. Additionally, ethanol enhances the permeability of the cell wall and promotes the decomposition of bacterial cells by dissolving fat-soluble substances on the cell wall. This process facilitates the penetration of n-hexane into the cellular matrix, allowing for more efficient extraction of lycopene (Zuorro, 2020; Nanou and Roukas, 2011; Coelho et al., 2022; Dhakane-Lad and Kar, 2021; Oberoi and Sogi, 2017; Strati and Oreopoulou, 2011).

### 3.2 Media optimization

#### 3.2.1 Single-factor experiment

**Figure 4:**
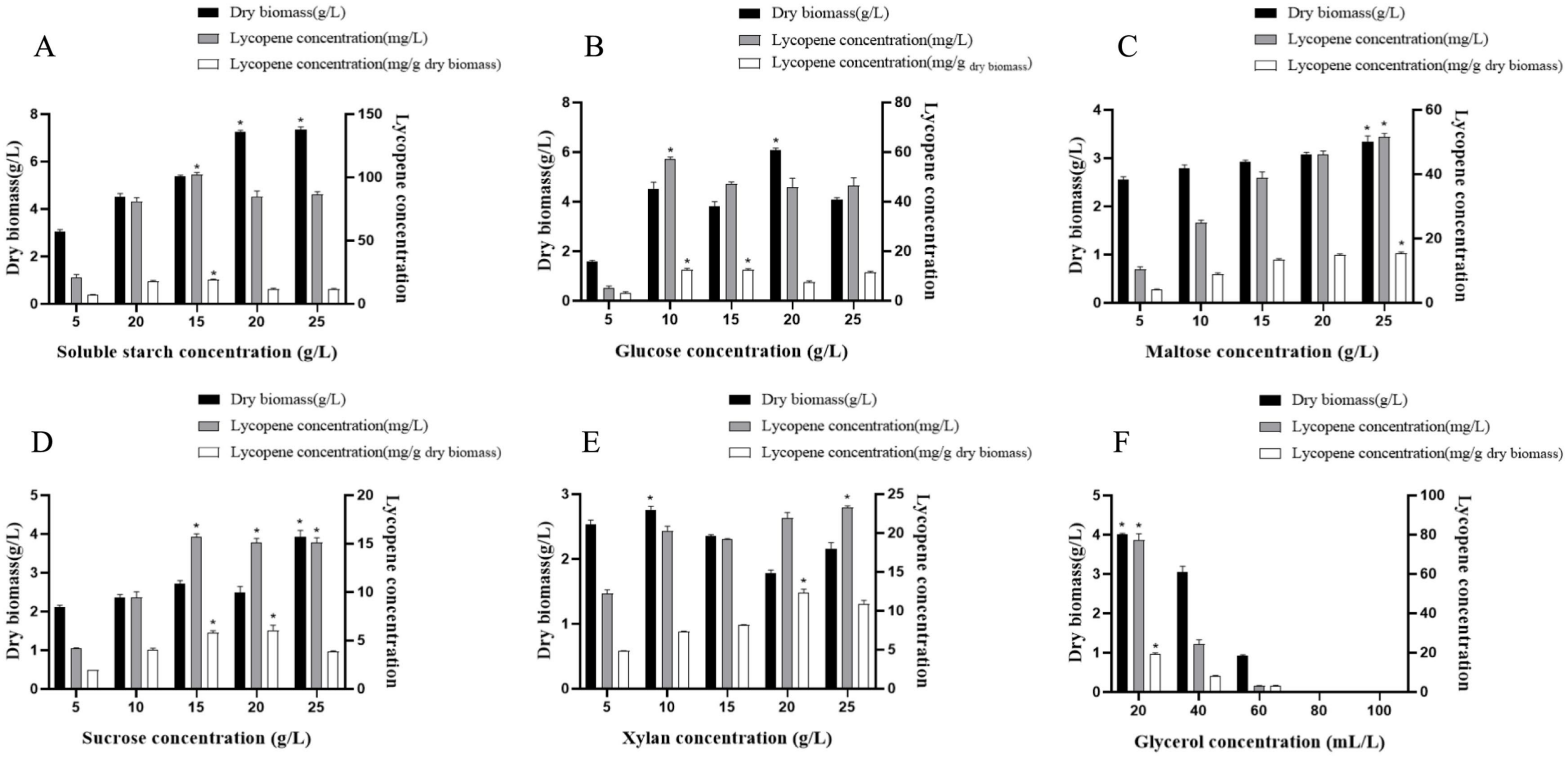
Histograms of dry biomass and lycopene yield of strain A560 after 7 days of cultivation at 28 °C in different carbon source media. **(A–E)** Represent soluble starch, glucose, maltose, sucrose, and xylan as the only carbon sources, respectively, and the concentrations range from 5 g/L to 25 g/L. **(F)** Represents the addition of different concentrations (20 mL/L–100 mL/L) of glycerol to ISP4 medium. The remaining sugar (g/L) was not quantified. The data are presented as mean ± SD from three independent experiments. ^⁎^*p* < 0.05.

Microbial growth and metabolite accumulation are significantly influenced by the component of the culture medium, as carbon sources influence both strain growth and target metabolite synthesis. This study investigated the effects of five carbon sources at different concentrations on the growth of strain A560 and the yield of lycopene synthesized by the fermentation of strain A560. Starch, a macromolecular carbohydrate, serves as a slow-release carbon source. It must be gradually hydrolyzed into monosaccharides by extracellular amylase, thereby avoiding the catabolite repression typically induced by high glucose concentrations. This sustained release of sugars enables cells to maintain metabolic activity and access utilizable carbon even during the later fermentation stages, ultimately enhancing the cumulative yield of lycopene (Liu and Shen, 2004). In this study, the highest level of lycopene production (102.49 ± 1.28 mg/L) was achieved with a soluble starch concentration of 15 g/L (Figure 4A and Supplementary Table S1). At starch concentrations ranging from 20 to 25 g/L, there was a concentration-dependent gradual decrease in lycopene yield, which aligns with findings reported in the literature (Hu et al., 2024). Fermentation using glucose, maltose, xylan, and sucrose as the sole carbon sources yielded maximum lycopene levels of 57.01 ± 0.96 mg/L, 51.60 ± 1.13 mg/L, 23.32 ± 1.18 mg/L, and 15.68 ± 0.95 mg/L, respectively (Figures 4B–E and Supplementary Tables S2–S5).

The selection and addition of oils significantly influence the stability and antioxidant properties of lycopene (Markham and Alper, 2018). Glycerol promotes lycopene accumulation in the cells by increasing solubility (Strati and Oreopoulou, 2011). The strain A560 exhibited notable differences in dry biomass and the yield of lycopene when it was cultured in ISP4 medium supplemented with varying concentrations of glycerol from 20 to 100 mL/L (Figure 4F and Supplementary Table S6). The strain grew vigorously in the medium supplemented with 20 mL/L glycerol, resulting in a dark orange culture solution. Under this condition, the dry biomass of the strain A560 was 4.00 ± 0.03 g/L, and the lycopene yield reached 77.41 ± 3.06 mg/L with a 25% improvement over the level of the control condition (62.05 ± 4.68 mg/L). The beneficial role of glycerol is likely twofold. First, glycerol serves as a hydrophilic solvent that improves the solubility of hydrophobic lycopene and mitigates its cytotoxicity (Wayama et al., 2013). Second, as a more reduced substrate rich in NADH, glycerol provides ample reducing power and favors lipid biosynthesis when nitrogen is limited. This metabolic redirection enhances the formation of lipid droplets, which, as reported, serve as storage sites for carotenoids and are linked to their biosynthetic pathway (Li et al., 2017). Thereby, glycerol indirectly promotes the accumulation and stability of lycopene within these hydrophobic compartments. However, with an increase in the concentration of glycerol, the cell biomass and the yield of lycopene decreased. When the concentration of glycerol reached 60 mL/L, the A560 strains did not grow due to the increase in osmotic pressure and cytotoxicity.

#### 3.2.2 Optimization results of a uniform design

Compared to an orthogonal design, UD conducts only one test for each factor and level, thereby reducing the number of tests and enhancing efficiency. In UD, the quantitative equation is generated by the computer, facilitating the analysis of how conditions affect the results and consequently minimize errors. Moreover, UD is particularly useful for studying factors with interactions. According to the single-factor test result of this study, adding an appropriate concentration of glycerol in the medium can enhance the solubility and stability of lycopene, resulting in the accumulation of lycopene in the strain A560. Therefore, glycerol was incorporated into the ISP4 medium, and a concentration gradient was designed for each fermentation medium component. The UD table of eight factors and nine levels was created using the software DPS 9.01 (Table 2).

**Table 2:** Experimental table of UD of fermentation medium for lycopene production from strain A560.

Groups	Factors							
	Soluble starch (g/L)	MgSO_4_^.^7H_2_O (g/L)	K_2_HPO_4_ (g/L)	NaCl (g/L)	CaCO_3_ (g/L)	(NH4)_2_SO_4_ (g/L)	Trace salt solution (mL/L)	Glycerol (mL/L)
N_1_	13	0.7	0.8	0.7	2.2	1.4	1.3	14
N_2_	9	1.4	0.7	0.9	2	2.2	0.6	16
N_3_	15	0.6	1.2	1.3	1.8	2.4	0.8	10
N_4_	21	0.8	0.6	1	1.4	2.8	1.1	20
N_5_	23	1.3	1.1	1.1	2.8	2	1.2	12
N_6_	19	1	0.9	1.2	2.4	1.2	0.7	26
N_7_	7	1.1	1	1.4	1.6	1.8	1.4	22
N_8_	17	1.2	1.3	0.6	1.2	1.6	0.9	18
N_9_	11	0.9	1.4	0.8	2.6	2.6	1	24

**Figure 5:**
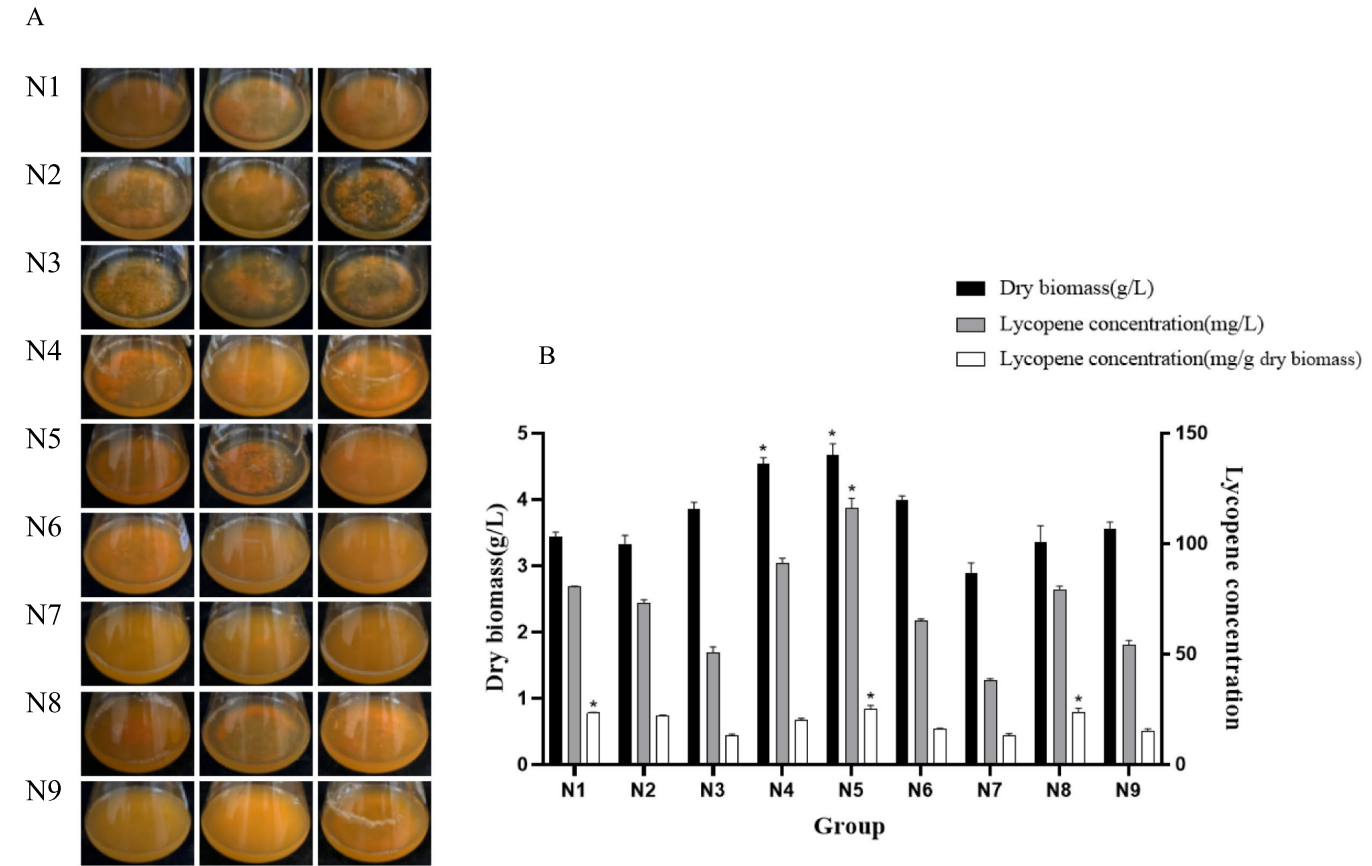
Growth state and lycopene yield of strain A560 after 7 days of incubation on the medium of each group of the UD. **(A)** State of the fermentation liquid. **(B)** Histogram of dry biomass and lycopene yield of strain A560. The remaining sugar (g/L) was not quantified. ^⁎^*p* < 0.05.

The culture media were prepared, and the strain A560 was inoculated into these media for fermentation to produce lycopene. The dry biomass and lycopene production were measured using the aforementioned method. The dry biomass of the fifth experiment group was 4.67 ± 0.17 g/L, and the lycopene yield reached 116.12 ± 4.43 mg/L, which was 87.14% higher than that of the basal medium (ISP4) (Figure 5). Compared with the base medium, the medium composition of the fifth experiment group was increased by 12 mL/L glycerol, the concentration of soluble starch increased by 130%, and the concentration of other inorganic salts increased slightly except for ammonium sulfate, indicating that the addition of glycerol promoted the accumulation of lycopene through utilizing soluble starch by strain A560.

A regression analysis was conducted using the software DPS 9.01 to derive a quadratic polynomial stepwise regression equation.

Y = 66.9411502 – 6803.098338×_4_^2^ + 864.4505932X_1_X_2_ + 101.91573279X_1_X_5_ + 12.776820504X_2_X_8_ + 500.5912850X_3_X_6_– 833.0808826X_3_X_8_ + 360.3413966X_7_X_8_

The correlation coefficient of the quadratic polynomial regression equation was *R*^2^ = 0.9999, where *Y* represents the predicted yield of lycopene and X_1_ to X_8_ represent the components of the medium, respectively. Based on the quadratic polynomial stepwise regression analysis, the theoretical optimal medium formulation (Ny) was determined as follows: soluble starch: 23 g/L, glycerol: 22 mL/L, CaCO₃: 2.8 g/L, (NH₄)₂SO₄: 2.8 g/L, MgSO₄: 1.4 g/L, trace salt solution: 1.4 mL/L, NaCl: 0.6 g/L, and K₂HPO₄: 0.6 g/L. The model predicted that the maximum yield of lycopene in a 250-mL conical bottle filled with 100 mL of liquid was 150.16 mg/L.

#### 3.2.3 Validation experiments

**Figure 6:**
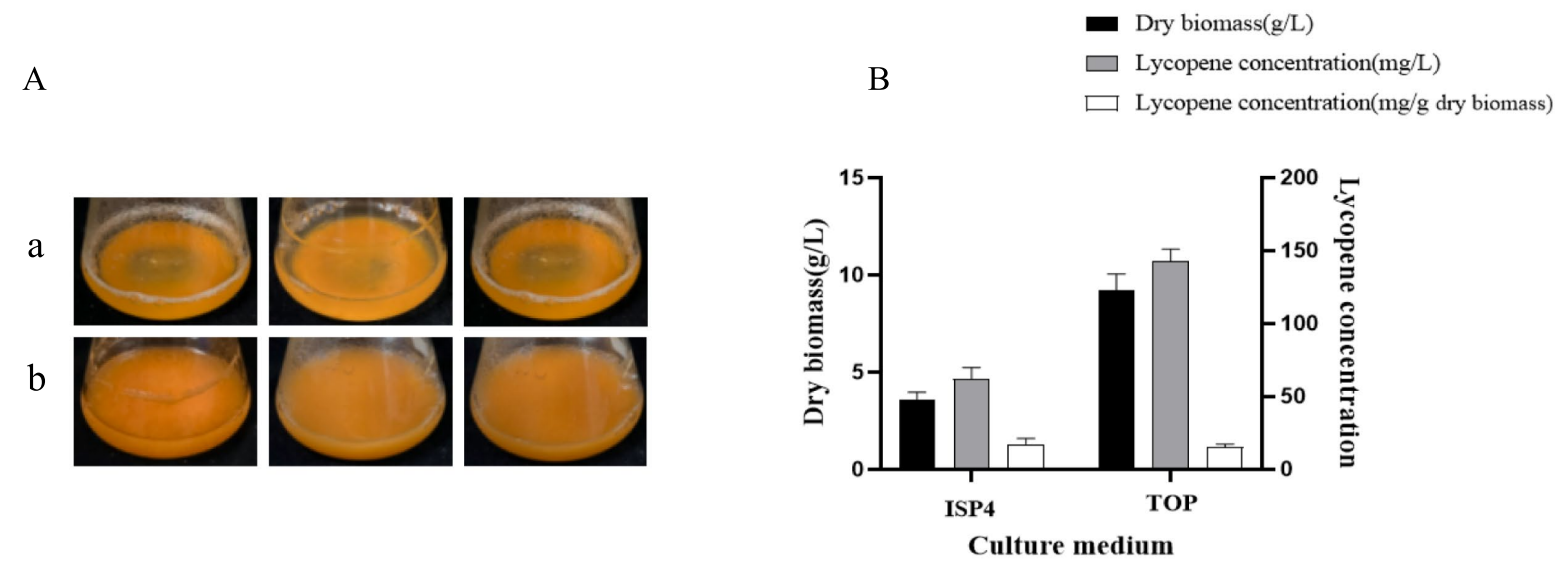
Growth state and lycopene yield of the strain A560 after 7 days of incubation on ISP4 medium and theoretically optimal medium. **(A)** State of the fermentation liquid: a. ISP4 medium, b. theoretically optimal medium. **(B)** Histogram of dry biomass and lycopene yield of strain A560. TOP, theoretically optimal medium. The remaining sugar (g/L) was not quantified.

The strain A560 was fermented using theoretically optimal medium and ISP4 medium under the above conditions. The results showed that the strain A560 grew well in the theoretically optimal medium (Figure 6A), and the dry biomass reached 9.23 ± 0.81 g/L. The lycopene obtained from the extraction of fermentation broth per liter was significantly enhanced with a yield of 142.5 ± 8.58 mg/L, which was 129.72% higher than that before optimization. Nevertheless, the amount of lycopene accumulated per gram of dried mycelium was reduced by 2 mg/g compared with that before optimization due to the large accumulation of cells in the late stage of fermentation and the reduction of available nutrients (Figure 6B).

### 3.3 Optimization of culture conditions

**Figure 7:**
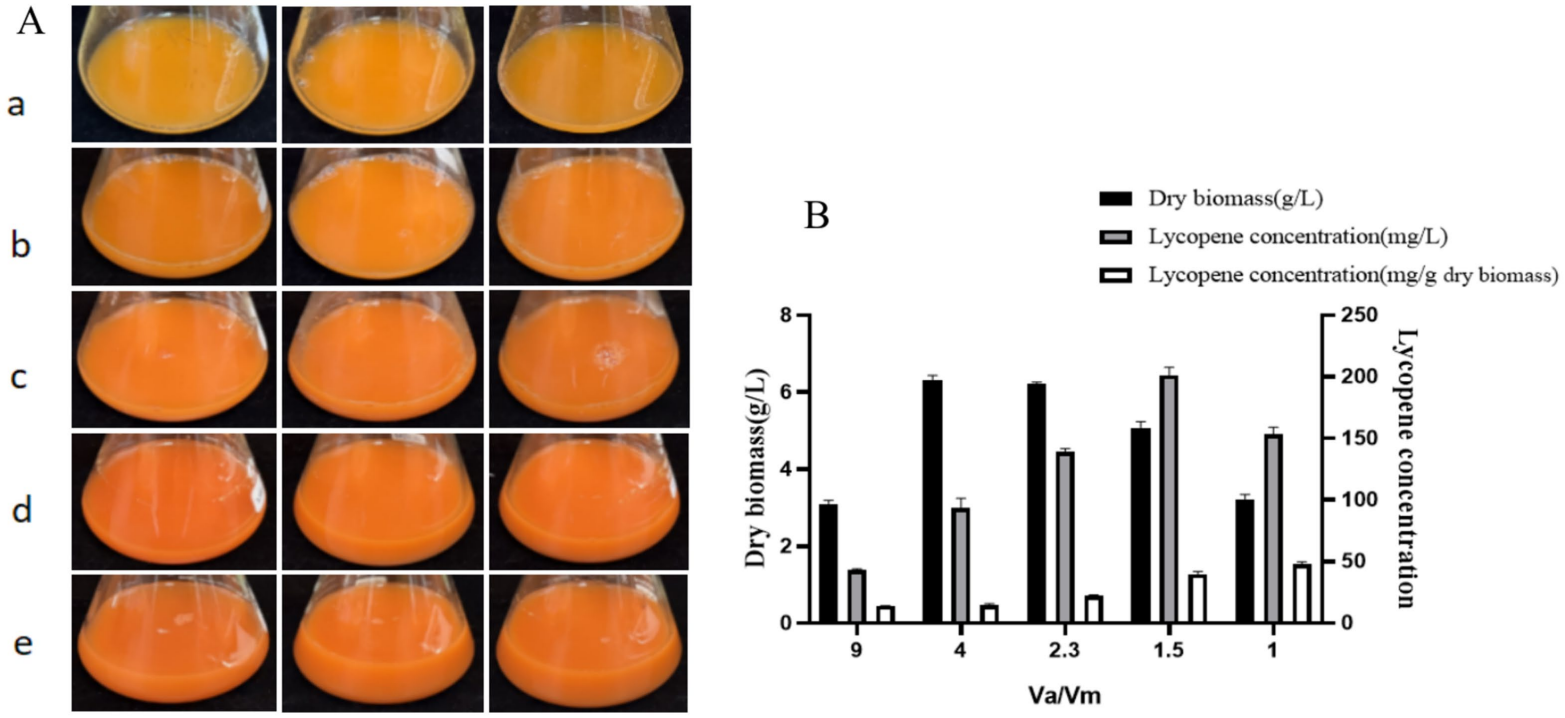
Growth and lycopene production of the strain A560 after 7 days of incubation on optimum medium with different liquid loading levels. **(A)** Fermentation broth: (a–e) represent the *Va/Vm* ratio of 9.0, 4.0, 2.3, 1.5, and 1.0, respectively. **(B)** Dry biomass and lycopene production of strain A560.

Lycopene production by microbial fermentation has been reported to be regulated by dissolved oxygen (DO) (Nanou and Roukas, 2011). One possible way to enhance lycopene yield is to expose cells to a low-oxygen environment. In this study, adjusting the *Va/Vm* ratio directly modulated DO in the fermentation medium: when the ratio was reduced from 9.0 to 4.0, DO was optimized for the growth of the strain A560, with biomass peaking at 6.33 ± 0.11 g/L. A further decrease in the *Va/Vm* ratio led to a decline in DO, accompanied by a gradual reduction in biomass (Figure 7B).

Beyond DO-mediated growth regulation, two additional factors contributed to the observed lycopene production dynamics. First, lycopene accumulation exhibited a contrasting trend to biomass under low DO conditions: at a *Va*/*Vm* ratio of 1.5 (nutrient-rich medium), lycopene yield reached a maximum of 201.44 ± 6.23 mg/L (a 224.64% increase compared with pre-optimization levels), which is consistent with the findings of Nanou and Roukas (2011). This peak in lycopene production under low DO and reduced biomass suggests that hypoxia redirects the limited available oxygen from primary metabolic pathways (supporting cell growth) to secondary metabolism (lycopene biosynthesis). Second, cytotoxicity from lycopene accumulation may further reduce bacterial biomass, as evidenced by the progressively darkening color of the fermentation broth with increased liquid loading (Figure 7A). Strain A560, a high-lycopene-producing actinomycete, harbors a comprehensive reactive oxygen species (ROS) detoxification system (including superoxide dismutase, catalase, and peroxidases), which may underpin its tolerance to low-oxygen stress and lycopene accumulation.

Additionally, low DO environments confer a dual benefit for lycopene production: in addition to channeling oxygen to biosynthesis, they suppress lycopene photodegradation, thereby enhancing product stability and reducing oxygen-induced cellular damage (Nishino et al., 2011). Collectively, low-oxygen conditions likely provide the minimal oxygen required for lycopene synthesis while activating antioxidant defense mechanisms that promote lycopene accumulation.

Table 3 summarizes the lycopene production performance of *Polymorphospora lycopeni* A560 in comparison with other microbial producers. Although the lycopene titer of A560 was lower than that of industrial high-yielding strains such as *B. trispora*, it exhibited distinctive advantages as a promising microbial resource.

**Table 3:** Comparison of lycopene production, fermentation conditions, and key strategies between *Polymorphospora lycopeni* A560 and other microorganisms.

Microbial strain	Strain characteristics	Fermentation conditions	Lycopene yield	Key strategy	Reference
*Polymorphospora* A560	Wild type	Shake flask fermentation	201.44 mg/L	Direct fermentation for metabolic synthesis without complex genetic engineering.	This study
*Blakeslea trispora*	Wild type	Shake flask fermentation and optimized diffused-bubble aeration fermentation.	256 mg/L	Fermentation optimization with a lycopene cyclase inhibitor.	Mantzouridou and Naziri (2017)
*Rhodobacter sphaeroides*	Recombinant Strain	Shake flask fermentation	10.32 mg/g _dry biomass_	Enhancing the MEP pathway, replacing *crtI*, and blocking the PPP pathway.	Su et al. (2018)
*Escherichia coli*	Recombinant strain	Fed-batch fermentation employing glucose and glycerol as carbon sources, under a two-stage temperature profile (33 °C initial, 28 °C later).	3.52 g/L	Enhanced by metabolic engineering through the introduction of heterologous *crtI* genes	Sun et al. (2014)
*Corynebacterium glutamicum*	Recombinant strain	Fed-batch fermentation	405.02 mg/L	The heterologous pathway was established by introducing the *crtE*, *crtB*, and *crtI* genes, while the competing pathway was disrupted by the knockout of *crtEb*.	Zhan et al. (2024)
*Saccharomyces cerevisiae*	Recombinant strain	120-h fed-batch fermentation (30 °C, pH 6.0, 1.5 vvm, DO ≥ 30%, 400–700 rpm) with feeding (glucose/yeast extract/ethanol) and galactose induction.	8.15 g/L	Introduction of the heterologous pathway by expressing the *crtE*, *crtB*, and *crtI* gene set from bacterial origin.	Zhou et al. (2023)
*Yarrowia lipolytica*	Recombinant strain	Fed-batch fermentation with glucose as the primary carbon source.	4.20 g/L	Introduction of the heterologous pathway by expressing the *crtE*, *crtB*, and *crtI* gene set from bacterial origin.	Luo et al. (2020)

Notably, A560 represents a novel actinomycete lycopene producer, expanding the diversity of lycopene-producing hosts beyond commonly reported fungi or engineered bacteria. As a natural producer, it requires no extensive genetic modification—unlike engineered *E. coli* or *Saccharomyces cerevisiae*—which simplifies industrial scale-up and enhances suitability for food and health products under strict regulatory frameworks. This strain serves as an excellent chassis with inherent lycopene biosynthesis capability. Its yield is expected to be significantly improved through metabolic engineering strategies successfully applied in *E. coli*, such as overexpression of rate-limiting genes or optimization of central carbon metabolism. Thus, the current moderate titer provides a solid foundation for future high-level production.

## 4 Conclusion

Currently, the strain used for industrial fermentation of lycopene production is primarily *Blakeslea trispora*. However, the fermentation process of lycopene produced by *B. trispora* is complicated, and the use of cyclase inhibitors increases the production cost, affects the safety of food, and increases the difficulty of downstream purification and extraction. In contrast, the strain *Polymorphospora lycopeni* A560 has the advantages of simple fermentation, high purity, and the absence of a requirement of a cyclase inhibitors.

In this study, the best method for extracting lycopene from the fermentation products of strain A560 was obtained; that is, n-hexane–ethanol (*v/v* = 2:1) was used as the extraction solvent. The optimal disruption parameters were determined as follows: homogenization at 4,500 rpm in cycles of 30 s disruption followed by 10 s pauses, repeated for a total duration of 4 min (PRECELLYS-24, Bertin), and then treated in a water bath at 60 °C for 1 h. At the same time, the optimal medium for lycopene production from strain A560 was obtained by a uniform design. A lycopene yield of 201.44 ± 6.23 mg/L was achieved by fermentation with the optimal medium at a volume ratio (*Va/Vm*) of 1.5. The optimization results of culture conditions showed that the appropriate reduction of dissolved oxygen was beneficial to the production of lycopene by A560 strain. This study provided the theoretical framework and experimental basis for the production of lycopene by industrial fermentation of strain A560.

## Data Availability

The original contributions presented in the study are included in the article/Supplementary material, further inquiries can be directed to the corresponding author.
