# Supplementary material for: Polymorphospora lycopeni A560, a new strain capable of producing lycopene, and its optimal fermentation and extraction conditions
Source: Front Microbiol. 2026 Jan 22;16:1723758. doi: 10.3389/fmicb.2025.1723758 (PMC12872836; doi:10.3389/fmicb.2025.1723758)
Supplement: Supplementary file 1 [file Data_Sheet_1.PDF]

Table S1: Dry biomass and lycopene yield of strain A560 after 7 days of incubation on medium with soluble starch as carbon source

Carbon source concentration (g/L)	Dry biomass (g/L)	Lycopene concentration (mg/L)	Lycopene concentration ($mg/g_{\text{dry biomass}}$)
5 g/L	3.06 ± 0.07[d]	21.14 ± 2.07[d]	6.91 ± 0.60[d]
10 g/L	3.78 ± 0.20[c]	61.87 ± 0.23[c]	16.32 ± 0.69[b]
15 g/L	5.37 ± 0.06[b]	102.49 ± 1.28[a]	19.08 ± 0.45[a]
20 g/L	7.25 ± 0.06[a]	84.52 ± 4.80[b]	11.65 ± 0.60[c]
25 g/L	7.34 ± 0.13[a]	86.51 ± 2.36[b]	11.79 ± 0.43[c]

[a] Different letters (a−d) imply significant differences ($p < 0.05$).

‘

Table S2: Dry biomass and lycopene yield of strain A560 after 7 days of incubation on medium with glucose as carbon source

Carbon source concentration (g/L)	Dry biomass (g/L)	Lycopene concentration (mg/L)	Lycopene concentration (mg/g dry biomass)
5 g/L	1.60 ± 0.04[e]	5.28 ± 0.60[c]	3.30 ± 0.38[e]
10 g/L	4.53 ± 0.25[b]	57.01 ± 0.96[a]	12.60 ± 0.53[a]
15 g/L	3.80 ± 0.20[d]	47.30 ± 0.63[b]	12.46 ± 0.50[a]
20 g/L	6.08 ± 0.08[a]	45.83 ± 3.58[b]	7.53 ± 0.49[c]
25 g/L	4.08 ± 0.08[c]	46.53 ± 3.08[b]	11.40 ± 0.54[b]

[a] Different letters (a–e) imply significant differences ($p < 0.05$).

Table S3: Dry biomass and lycopene yield of strain A560 after 7 days of incubation on medium with maltose as carbon source

Carbon source concentration (g/L)	Dry biomass (g/L)	Lycopene concentration (mg/L)	Lycopene concentration (mg/g dry biomass)
5 g/L	2.55 ± 0.06[e]	10.39 ± 1.71[e]	4.06 ± 0.67[d]
10 g/L	2.79 ± 0.07[d]	24.84 ± 1.82[d]	8.80 ± 1.84[c]
15 g/L	2.92 ± 0.04[c]	38.96 ± 3.02[c]	13.34 ± 0.88[b]
20 g/L	3.08 ± 0.04[b]	46.03 ± 4.08[b]	14.85 ± 1.53[a]
25 g/L	3.35 ± 0.11[a]	51.60 ± 1.13[a]	15.42 ± 1.26[a]

[a] Different letters (a–e) imply significant differences ($p < 0.05$).

Table S4: Dry biomass and lycopene yield of strain A560 after 7 days of incubation on medium with sucrose as carbon source

Carbon source concentration (g/L)	Dry biomass (g/L)	Lycopene concentration (mg/L)	Lycopene concentration (mg/g dry biomass)
5 g/L	2.12 ± 0.04[d]	4.19 ± 0.11[c]	1.97 ± 0.44[c]
10 g/L	2.36 ± 0.08[cd]	9.45 ± 0.58[b]	4.00 ± 0.62[b]
15 g/L	2.71 ± 0.20[b]	15.68 ± 0.95[a]	5.78 ± 0.20[a]
20 g/L	2.49 ± 0.29[bc]	15.09 ± 1.35[a]	6.08 ± 0.51[a]
25 g/L	3.92 ± 0.17[a]	15.11 ± 0.80[a]	3.86 ± 0.40[b]

[a] Different letters (a–d) imply significant differences ($p < 0.05$).

Table S5: Dry biomass and lycopene yield of strain A560 after 7 days of incubation on medium with xylan as carbon source

Carbon source concentration (g/L)	Dry biomass (g/L)	Lycopene concentration (mg/L)	Lycopene concentration (mg/g dry biomass)
5 g/L	2.53 ± 0.15[b]	12.24 ± 1.21[e]	4.84 ± 0.33[e]
10 g/L	2.76 ± 0.05[a]	20.22 ± 1.49[d]	7.34 ± 1.13[d]
15 g/L	2.35 ± 0.02[c]	19.18 ± 0.13[c]	8.15 ± 0.47[c]
20 g/L	1.79 ± 0.10[e]	21.96 ± 0.67[b]	12.29 ± 1.12[a]
25 g/L	2.15 ± 0.10[d]	23.32 ± 1.18[a]	10.85 ± 0.98[b]

[a] Different letters (a–e) imply significant differences ($p < 0.05$).

Table S6: Represents addition of different concentrations (20 mL/L-100 mL/L) of glycerol to ISP4 medium.

Carbon source concentration (mL)	Dry biomass (g/L)	Lycopene concentration (mg/L)	Lycopene concentration (mg/g dry biomass)
20 mL/L	4.00 ± 0.03^{a}	77.41 ± 3.06^{a}	19.33 ± 0.64^{a}
40 mL/L	3.06 ± 0.14^{b}	24.41 ± 2.18^{b}	7.98 ± 0.36^{b}
60 mL/L	0.92 ± 0.03^{c}	3.03 ± 0.15^{c}	3.28 ± 0.08^{c}
80 mL/L	0	0	0
100 mL/L	0	0	0

[a] Different letters (a–c) imply significant differences ($p < 0.05$).
